# Broadband Solar Absorber Based on Square Ring cross Arrays of ZnS

**DOI:** 10.3390/mi12080909

**Published:** 2021-07-30

**Authors:** Feng Xu, Lixia Lin, Jun Fang, Mianli Huang, Feng Wang, Jianzhi Su, Shufen Li, Miao Pan

**Affiliations:** 1 College of Chemical Engineering and Materials Science, Quanzhou Normal University, Quanzhou 362000, China; Fengxu202101@163.com (F.X.); 13774559778@163.com (L.L.); fangjun@qztc.edu.cn (J.F.); mianlihuang@qztc.edu.cn (M.H.); 2 College of Physics & Information Engineering, Quanzhou Normal University, Quanzhou 362000, China; fwang99@126.com (F.W.); sujianzhi@126.com (J.S.); shufenli55@163.com (S.L.)

**Keywords:** solar absorber, broadband, perfect absorption, surface plasmon

## Abstract

Solar energy is an inexhaustible clean energy. However, how to improve the absorption efficiency in the visible band is a long-term problem for researchers. Therefore, an electromagnetic wave absorber with an ultra-long absorption spectrum has been widely considered by researchers of optoelectronic materials. A kind of absorbing material based on ZnS material is presented in this paper. Our purpose is for the absorber to achieve a good and wide spectrum of visible light absorption performance. In the wide spectrum band (553.0 THz–793.0 THz) of the absorption spectrum, the average absorption rate of the absorber is above 94%. Using surface plasmon resonance (SPR) and gap surface plasmon mode, the metamaterial absorber was studied in visible light. In particular, the absorber is insensitive to both electric and magnetic absorption. The absorber can operate in complex electromagnetic environments and at high temperatures. This is because the absorber is made of refractory metals. Finally, we discuss and analyze the influence of the parameters regulating the absorber on the absorber absorption efficiency. We have tried to explain why the absorber can produce wideband absorption.

## 1. Introduction

One of the major issues that are limiting the development of human society is the energy crisis. The most promising new green energy source for the twenty-first century is solar energy. One way to effectively alleviate the energy crisis is to make full use of solar energy. We know that broadband absorption close to the solar spectrum is necessary to effectively collect and use solar energy. Plasmon absorbers with unique subwavelength trapping capability have received special attention from researchers [1,2,3,4]. In 2008, Landy et al. proposed an electromagnetic wave absorber, which attracted the attention of researchers [5]. We observe visible light on Earth at frequencies ranging from 380 THz to 850 THz. The best way of obtaining solar energy is through solar thermal systems [6]. A perfectly designed absorber is the key to a solar thermal system being able to absorb solar radiation perfectly. An ideal absorber should have a sufficiently broad absorption curve with good absorption performance in the visible wavelength band. The absorber should also be insensitive to electric field absorption and magnetic field absorption [7,8,9,10]. We combine multiple surface pattern structures in the absorption layer of the same resonant cell to achieve broadband absorption [11,12,13,14]. This multi-resonance combination method can make multiple resonance absorption peaks in the spectrum overlap with each other. We end up with a wide absorption bandwidth [15,16]. However, the broadband of the absorber cannot be infinitely wide due to the influence of adjacent resonant arrays and the fact that an array of cells can only accommodate a limited combination of resonator stacks [17]. Au can produce plasmon resonance and optical coupling. Therefore, Au is used in the metal material of the absorber designed in this paper [18].

Metamaterials have unique absorption properties. Using metamaterials to design ideal absorbing materials has become the focus of researchers [19,20,21,22]. Metamaterials can completely absorb microwaves. Metal–insulator–metal (MIM) consists of three parts of a plasmon absorber. The stack structure of the absorber can accomplish multi-band spectral absorption or wide spectrum absorption [23,24,25,26,27,28]. The top layer of the metal–insulator–metal absorber is composed of a metal array pattern, the middle layer is a dielectric layer, and the bottom is a metal substrate to prevent transmission. The bottom metal film is used to prevent the transmission of electromagnetic waves. The patterned metal structure on the top layer is used to match the spatial impedance and suppress the reflection of electromagnetic waves. In addition, the ability of metamaterial absorbers to absorb light depends not only on the material itself but also on its shape, size, arrangement, and structure. Many researchers have designed solar absorbers based on membrane stacking, but these absorbers usually have very narrow absorption bands [29,30,31]. In 2017, Luo et al. developed a sandwich structure metamaterial based on nickel (Ni) film [32]. In this metamaterial structure, SPP resonance is excited at the interface of Ni film and air. A resonant cavity mode exists in the grooves of the absorber. The integration of the SPP syntony and the resonator mode results in a wide band response. Ni has a strong absorption property in the visible band. Therefore, the sandwich metamaterial structure has perfect absorption performance in the whole visible light band. In 2017, Cao et al. realized the perfect absorption of the whole visible band by using Ge2Sb2Te5 material with a large imaginary part of dielectric function in the visible band [33]. Takatori et al. developed a silver-based broadband absorber. The average absorption rate of the silver-based absorber is more than 50% in the wavelength range of 400 nm–3200 nm [34]. The combination of surface plasmon resonance and resonant cavity mode can localize the electromagnetic field in the dielectric gap to achieve near-perfect absorption [35,36,37,38].

Inspired by the literature published by Luo in 2017, we decided to adopt the stack structure of a thin film layer to achieve better absorption efficiency in the visible light band. Inspired by Cao et al.’s literature in 2017, we used refractory precious metal gold to replace silver material so that the absorber can still maintain a high absorption rate at high temperatures. The former work uses the combination of surface plasmon resonance (SPR) and cavity mode to enhance absorption. Therefore, this paper also designed the structure to make the surface plasmon resonance and resonant cavity mode obtain better absorption efficiency. ZnS material is an important II–VI compound semiconductor. ZnS material is a kind of common wide bandgap semiconductor material. ZnS nanomaterials have attracted much attention. In this paper, a broadband absorber with a multilayer structure based on ZnS material is proposed. The structure of the absorber and the materials used can interact to produce surface plasmon resonance. Surface plasmon resonance results in strong absorption or scattering of incident photons on the metal surface. The absorber concentrates the energy of the electromagnetic field in the subwavelength range, so the absorber produces a very strong light field enhancement effect. This effect greatly increases the interaction between light waves and absorbent materials. Therefore, we could obtain an absorber with good performance and a wide spectrum. Notably, the multilayer metamaterial achieved an average good absorption rate of 94% across the 240 THz spectrum, with a single peak of up to 99.7% at 396.0 THz. In addition, the absorptivity is insensitive to polarized light. The absorber based on precious metal will deform at a relatively low temperature. Strong plasmon resonance can enhance the absorption of light. When the structure is exposed to strong light, the structure of the absorber based on precious metal may deform and lose its original absorption performance. The UWB absorber we proposed is based on refractory metal and semiconductors, which can maintain a stable structure at high temperatures [39]. Therefore, our proposed ultra-wideband electromagnetic wave absorber has wide application foreground in the fields of thermal photovoltaic, transducer, cloaking, and infrared detection [40,41,42,43,44]. At the same time, this paper can provide some suggestions for the design of the absorber of the film stack structure.

## 2. Design and Structure

**Figure 1:**
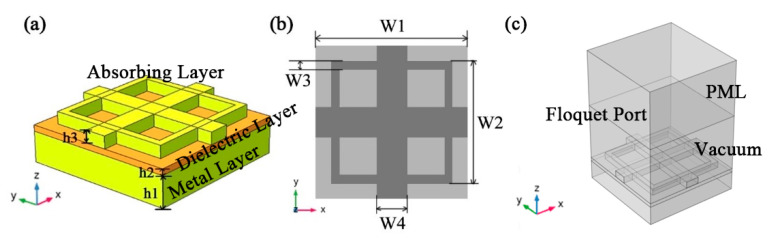
Schematic diagram of the base absorber: (**a**) schematic diagram of the three-dimensional structure of the ultra-broadband solar absorber. (**b**) Top view of the absorber structure. (c) the perfect matching layer (PML) of the base absorber.

Figure 1 shows the structure of the absorber. Each cell array consists of three layers stacked on top of each other [45,46,47]. The top layer is composed of an Au microstructure layer. The dielectric function of Au film is the Drude model [48]. The structural parameters of the metamaterial are shown in Figure 1. The period width of the absorber is W1 = 1000 nm, and the width of the square ring structure is W2 = 800 nm, the square ring spacing is W3 = 100 nm, the cross-structure width is W4 = 140 nm, and the thickness of the absorption layer is h3 = 80 nm. A semiconductor material ZnS is used as the dielectric layer in the middle, and its thickness is h2 = 40 nm. We use the Au layer with h1 = 200 nm thickness at the bottom to eliminate the transmission in order to make the absorber transmittance zero. The total thickness of the whole nanocavity is 320 nm. COMSOL software was used to verify whether the absorber proposed has a good absorption effect [49]. In this paper, the absorption characteristics of square cross multilayer metamaterials are simulated by using the finite element method. The boundary conditions during simulation are set as follows. The top layer of the air layer outside the absorber is modeled with the perfect matching layer and the scattering boundary conditions. The goal is to allow as much plane light with frequencies between 380 THz and 850 THz to shine vertically into the absorber from above the *z*-axis. Since the absorber is composed of multiple repeated cell arrays, we set Floquet periodic boundary conditions around the cell arrays. The research of an array of cells in the absorber can reduce the computational burden.

## 3. Results and Discussion

**Figure 2:**
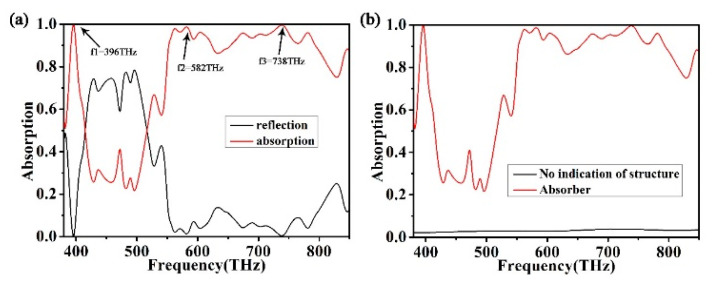
(**a**) Ultra-broadband spectrum of a solar absorber. (**b**) Spectrum of an absorber without surface structure.

As the thickness of Au is much larger than that of the substrate through which the electromagnetic wave can penetrate, the transmittance of the structure is basically zero. The absorption spectrum of multilayer stacked metamaterials can be calculated by A = 1 − R [50,51,52,53,54]. As shown in Figure 2a, the absorption spectrum is marked by red lines, and the reflectivity is represented by black lines. As can be seen from Figure 2a, there is one peak in the low-frequency part and two peaks in the high-frequency broad-spectrum absorption part. The three different peaks are 396 THz, 582 THz, and 738 THz, and their respective absorptions are 99.67%, 98.71%, and 99.61%, respectively. Therefore, the structure we researched has wider and better absorption lines. As shown in Figure 2b, we compare the absorption spectra with and without the top antireflection layer. When there is an antireflective layer on the top, the absorber has better absorption performance. The absorbency of the absorber is more than 90% in the wavelength range of 240 THz, with an average absorbency of 94%. There are two maximum absorption peaks, f2 and f3, in the broad spectrum, and the absorptions are 98.71% and 99.61%, respectively. There is a single peak f1 with perfect absorption in the low-frequency part, and the maximum absorption is 99.7%. When there is no absorption layer, we can see from Figure 2b that the absorption efficiency of the absorber to visible light is very low. The results show that the uppermost structure contributes the most to light absorption.

To evaluate the performance excellence of the proposed structure, we compare the results with other similar absorbers, as shown in Table 1. Obviously, our proposed absorber has a higher average absorption rate in the visible band

**Figure 3:**
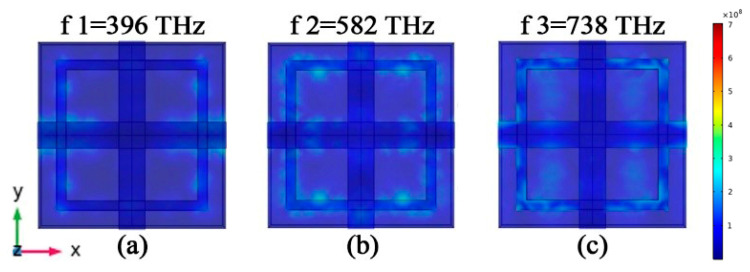
(**a**) Electric field diagram for a frequency of 396 THz. (**b**) Electric field diagram for a frequency of 582 THz. (**c**) Electric field diagram for a frequency of 738 THz.

**Figure 4:**
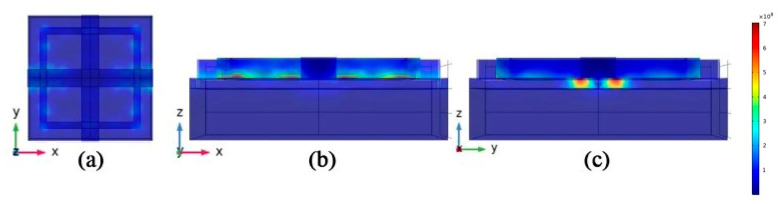
(**a**) top view of the electric field at a frequency of 396 THz. (**b**) Electric field view from the *y*-axis direction; (**c**) electric field view from the *x*-axis direction.

We simulated the electric field distribution at absorption peaks f1, f2, and f3, in order to understand why the absorber can absorb solar radiation in the visible band. We know that the SPP response can be excited at the metal–dielectric interface by the incident electromagnetic wave. The MIM structure has two metal–dielectric interfaces, and the two SPP modes are coupled in the dielectric layer to form the interstitial surface plasmon mode. The gap surface plasmon mode is derived from the coupling of two SPP modes. Therefore, the gap surface plasmon modes propagate along the polarization direction. The propagation of this mode is limited at the *z* axis. A schematic diagram of electric field in X–Y plane is shown in Figure 3a–c. The frequency of absorption peak f1 is 396 THz. The gap surface plasmon modes propagate along the y-axis direction. When the surface structure size meets certain conditions, the gap surface plasmon modes propagate along the y-axis in both forward and backward directions. The absorber will produce a perfect absorption peak due to the standing wave syntony of a specific wavelength. The frequency of the absorption peak f2 is 582 THz, and the absorption excited by the electric field is distributed in the *y*-axis direction of the pattern layer on the top of the absorber. The distribution of the electric field enhances the absorption between the absorbing layer and the dielectric layer. Absorption peak f3 is located at 738 THz. The electric field distribution is similar to that of f2. As can be seen from Figure 4a, each cell array in the absorption layer excites surface plasmon between the top Au layer and the ZnS layer. The results show that the incident electromagnetic wave excites the SPP and forms the gap surface iso-polariton mode on the absorber, which enhances the light absorption. These electric fields are tightly confined between the metal and the medium. From Figure 4b,c, we can conclude that the absorption enhancement excited by the electric field is concentrated in the *y*-axis direction of the absorption layer. In the *x* direction, the incident electric field of the absorber is mainly confined to the dielectric layer. In summary, the square ring cross absorber designed in this paper can achieve broad-spectrum light absorption mainly by excitation of the surface plasmon and interstitial surface plasmon mode. The absorber relies on standing wave resonance to achieve a strong electric field localization effect to achieve the perfect absorption peak at f1.

**Figure 5:**
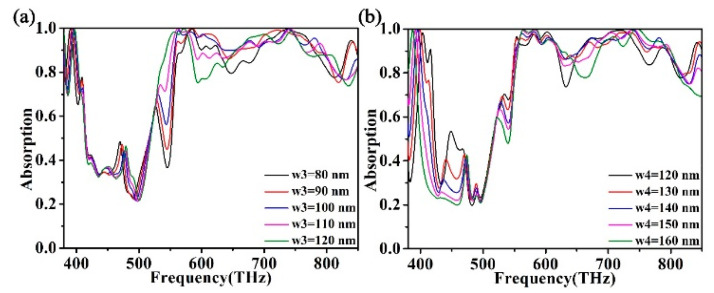
(**a**) The effect of square ring spacing W3 on the absorption spectrum. (**b**) The effect of cross-width W4 on the absorption spectrum.

The aim of this study is to investigate whether changing the structural parameters affects the performance of the absorber. An attempt was made to adjust the structural parameters of the metamaterial absorber. We adjusted the spacing of the top square ring structure W3. The absorption effect is shown in Figure 5a. We changed the distance W3 of the square ring structure of the absorber from 80 nm to 120 nm in steps of 10 nm. The absorption peak of the absorber increased from 83.3% to 99.59% at f1 and from 92.0% to 99.1% at f2. The absorption performance of the absorber is significantly improved. While the absorption peak of f3 fluctuated slightly, the absorption of the absorber at the frequency of f3 first dropped from 99.4% to 98.2% and then rose to 99.4%. The absorption band of the absorber gradually widened, and the absorption rate between f1 and f2 also increased to more than 87.5%. The reason for these phenomena is that ZnS nanometer resonators provide effective resonance absorption through square ring resonance and cross plasmon resonance [60,61]. Therefore, in order to obtain an ideal absorption peak, appropriate square ring resonance and cross resonance should be adjusted to maximize the superposition resonance absorption. The superposition of absorption peaks produces ultra-wideband absorption. At f1 and f2, the resonance of the absorber is significantly enhanced with the decrease of W3. The resonance reaches the maximum at W3 = 100 nm. We can observe and conclude from Figure 5b that as W4 increases, the absorption peak of the absorber at f2 also increases. The absorptivity of the absorber increased from 97.34% to 99.85%. When W4 = 160 nm, the resonance absorption of the absorber reaches the maximum, and the absorption efficiency graph is red-shifted. The resonance of the absorber reaches the maximum at W4 = 150 nm, and the absorption peak at f3 reaches 99.85%. When W4 = 150 nm, the absorbance of f3 is as high as 99.9%, but the absorption efficiency of the absorber is not as wide as that of W4 = 140 nm. In addition, we can also observe that the structural parameters change in a wide range, and the absorber can still maintain a wide absorption bandwidth and high absorption efficiency. These characteristics will be beneficial to physical manufacturing because the absorber has a high tolerance.

**Figure 6:**
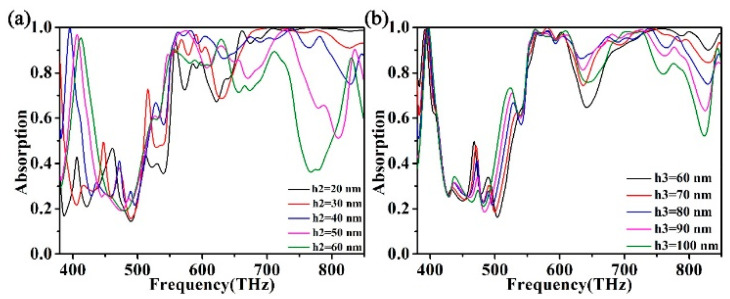
(**a**) The effect of dielectric layer thickness H2 on the absorption spectrum. (**b**) The effect of surface absorption layer thickness H3 on the absorption spectrum.

Afterward, we wanted to find out whether changes in the thickness of the film would have an effect on the absorption spectrum. We changed the ZnS film thickness h2 and Au film thickness h3 of the absorber. Observing Figure 6a, it can be concluded that as the ZnS film thickness h2 increases, the absorber appears red-shifted, and the overall absorption efficiency moves towards the lower frequencies. The absorption peak of the absorber at f1 fluctuates greatly. The absorption efficiency of the absorber increased from 24.0% to 99.68% and then decreased to 55.94% due to the red-shifted of the absorption peak at f1. The absorption peaks of the absorber begin to split at f2 and f3. The absorption efficiency between f2 and f3 is significantly reduced. The absorption effect of the high-frequency part of the absorber decreased significantly. When h2 is greater than 40 nm, the absorption effect of the absorber between f2 and f3 begins to improve again. In summary, when h2 is adjusted to an appropriate thickness, a wide spectrum of absorption can be formed between f2 and f3. When the thickness of ZnS film h2 is set at about 40 nm, the absorption intensity of absorption peaks at f2 and f3 has no obvious change. By observing Figure 6b, we can see that the absorption map of the absorber is red-shifted after the change of the film thickness of the absorbing Au layer h3. With the augment of Au layer thickness, the absorption rate of the absorber at f1 increases from 90.0% to 99.7%. This phenomenon indicates that resonant light becomes stronger and stronger in the cavity [62,63]. However, the absorption efficiency between f2 and f3 increased significantly, but the absorption efficiency of the high-frequency part after f3 decreased gradually. This is because the resonance enhancement at f2 and f3 results in the red-shifted absorption peak at f3 from 738 THz to 702 THz. Obtaining the results of changing the structural parameters in Figure 6a,b, we found that the size of the absorber structure has a great effect on the absorber’s performance. Therefore, the selection of appropriate absorber layer thickness h3 and dielectric layer thickness h2 is crucial to whether the absorber can achieve perfect absorption of solar radiation.

**Figure 7:**
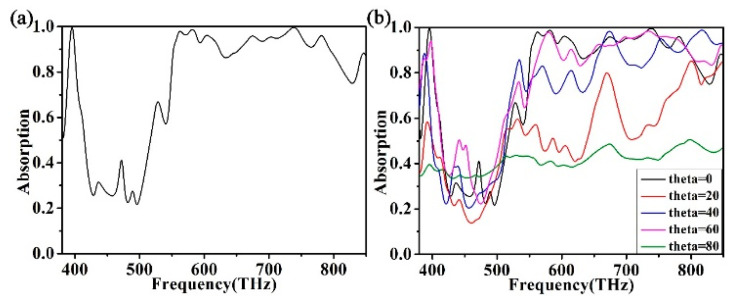
(**a**) The absorption curve under TM polarization (transverse magnetic, the electric field is parallel to the *x* direction). (**b**) The absorption spectrum of the absorber at different angles of incidence.

It is well known that visible light does not necessarily incident vertically in practical applications. Insensitive to both polarization and angle of incidence, it is an ideal absorber in the minds of researchers. We varied the pitch angle and polarization of the incident light in order to investigate whether the angle of incidence and polarization had an effect on the absorption spectrum of the absorber. We investigated the effect of different polarized light and incident angles on the absorption spectra. The absorption spectrum of the absorber does not change significantly when the incident magnetic field changes to the incident electric field. The absorption spectrum when the incident field is a magnetic field is shown in Figure 7a. Therefore, the absorber is insensitive to TE and TM light. This conclusion is primarily caused by the symmetrical arrangement of the square ring cross structure in the periodic array. The absorption of the absorber at 0–80° incidence is shown in Figure 7b. When the back oblique incidence angle is 40° and 60°, the absorption of the absorber begins to decrease in part of the frequency range. The incident light from different angles has an effect on the absorption performance of the absorber. The results suggest that the effect is limited. The calculation results show that the wideband absorber has strong angle sensitivity. The absorptance of the absorber decreases as the angle of incidence increases. This is because as the angle of incidence increases, the component of the incident magnetic field in the *x*-axis direction decreases. The decreasing × component causes the magnetic flux density between the surface metal blocks to become smaller and smaller. Less and less energy is being absorbed into the absorber. As a result, there will be less and less electromagnetic absorption [64,65]. Despite the increase in the angle of incidence to 60°, the absorber still has a high absorption rate. The absorber can still meet the requirements of many practical applications.

**Figure 8:**
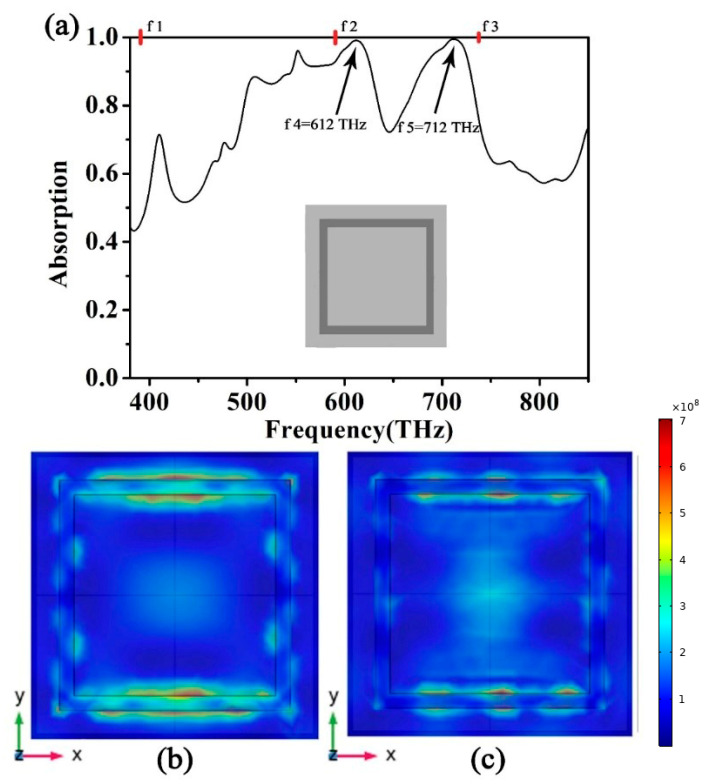
(**a**) Absorption spectrum of the absorber in the presence of a magnetic field with only a square ring structure. (**b**) The electric field diagram of the absorber at f4. (**c**) The electric field diagram of the absorber at f5.

**Figure 9:**
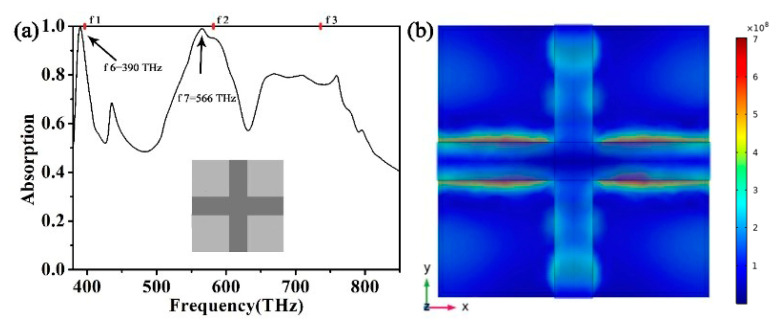
(**a**) Absorption spectrum of the absorber in the presence of a magnetic field with only the cross structure. (**b**) The electric field diagram of the absorber at f6.

**Figure 10:**
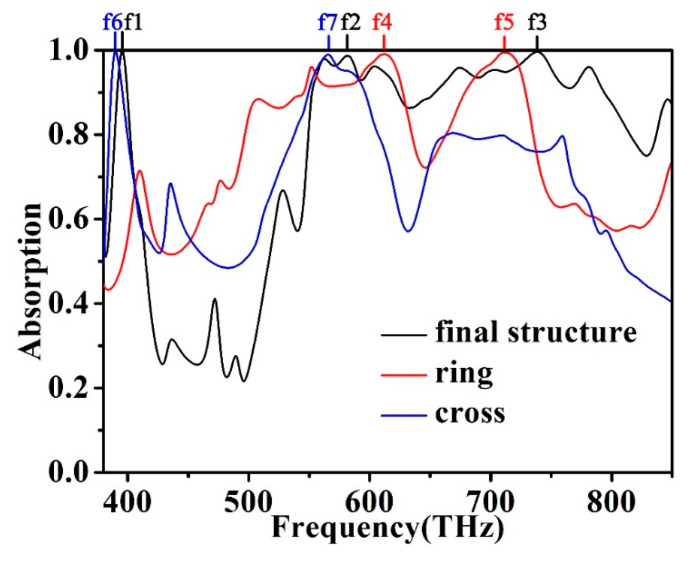
The absorption of the ring, the cross, and the final structure.

Finally, we want to understand how each part of the absorber affects the performance of the absorber, and we divided the proposed absorber into two parts for research. We only kept the top square ring structure of the absorber. The absorption spectrum and the top structure are shown in Figure 8a. We can find that there are two better absorption peaks, f4 = 612 THz and f5 = 712 THz, and their absorptions are 99.1% and 99.5%, respectively. This result confirms that the wide spectrum absorption caused by the excited surface plasmons is mainly provided by the square ring structure. Figure 9a shows the absorption spectrum of the absorber with only a cross structure. We can find that there are two better absorption peaks, f6 = 390 THz and f7 = 566 THz, and their absorptions are 99.7% and 99.0%, respectively. The results confirm that the ideal single absorption peak in the low-frequency part is mainly provided by the cross structure when the two structures become the metamaterial absorber proposed in this paper. Due to the superposition of the absorption properties of f4 and f7, we finally obtain the absorption efficiency of the absorber at f2. The ideal absorption peak of the absorber at f6 is also transferred to f1 due to the superposition of absorption properties. The ideal absorption peak of the absorber at f5 is also transferred to f3. This conclusion is shown in Figure 10. After the cross and square ring are superimposed, the absorption pattern changes from four absorption peaks to three absorption peaks. Due to the interaction of these two structures, we obtained a metamaterial absorber with an ideal absorption peak and a wide spectrum of visible light bands. The design of axisymmetric structures insensitive to polarization is a method to significantly enhance surface plasmon resonance. The final effect of surface plasmon resonance, therefore, rests with the mode structure of the absorbing layer at the top of the metamaterial. By reason of the foregoing, the absorber designed in this paper has a better effect than other absorbers that absorb visible light radiation.

## 4. Conclusions

In this paper, a wideband absorber with a stacked membrane structure is designed. Each array element of the absorber is a sandwich structure consisting of a top absorber layer with a square ring cross pattern, a dielectric layer made of ZnS, and a bottom film made of Au. The absorber has an absorbance of more than 90% in the visible wavelength range of 240 THz, with an average absorbance of 94%. There are two maximum absorption peaks in the wide spectrum, f2 and f3, with absorption rates of 98.71% and 99.61%, respectively. There is a perfectly absorbed unimodal f1 in the low-frequency section, with a maximum absorption rate of 99.7%. Wideband absorption is primarily caused by surface plasmon resonance and gap surface plasmon mode. On this basis, the effects of different structural parameters on the absorption characteristics of the absorber are studied in detail. The ultra-wideband absorption of the perfect absorber proposed in this paper is polarization-independent. This characteristic allows the absorber to have a good performance of electromagnetic wave absorption under electromagnetic conditions. The material we used has a high thermal stability, which indicates that the absorber has a potential application prospect in high-intensity irradiation and high temperatures. The broadband absorber proposed in this paper has a broad application prospect in solar photovoltaic power generation, stealth, thermal electronic equipment, and other fields.

## Figures and Tables

**Table 1:** Comparison with other similar visible band absorbers.

Refer.	Absorption Band-Width	Modulation Depth	The Average Absorption
This article	553 THz–793 THz	Over 90%	94%
[55]	523–592.5 THz	Over 90%	<90%
[56]	375 THz–750 THz	Over 90%	70%
[57]	481.2 THz–684.0 THz	Over 90%	92%
[58]	430 THz–770 THz	Over 90%	93.7%
[59]	400 THz–750 THz (N = 2)	Over 90%	90%
